# Supplementary material for: Identification of Freezing-Responsive microRNAs and Their Targets in Chinese Jujube by Small RNA and Degradome Sequencing
Source: Int J Mol Sci. 2026 Sep 4;27(17):7912. doi: 10.3390/ijms27177912 (PMC13566890; doi:10.3390/ijms27177912)
Supplement: Supplementary file 1 [file ijms-27-07912-s001.zip › Supplementary Material S2/novel_miR_168.pdf]

Provisional ID	:	novel_miR_168
Score total	:	3.5
Score for star read(s)	:	-1.3
Score for read counts	:	0
Score for mfe	:	3.2
Score for randfold	:	1.6
Score for cons. seed	:	
Total read count	:	2250
Mature read count	:	1178
Loop read count	:	929
Star read count	:	143

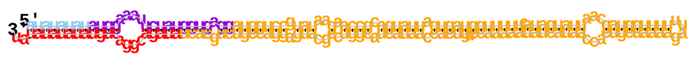

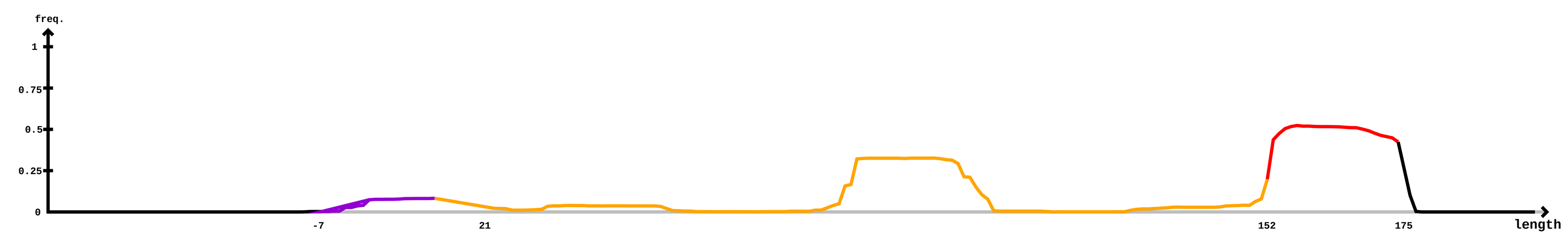

Star

Mature

```
5'- cuuuuauauggugugccaaagccauucuuaacacauauauuuauauauauauauggaaauucuauggugaggaugguccguauaagaaccgcaguauuagugaugguuuuucauaguauuaacgaugguuuuuuagaaaaucgucaccaauacuauaaaaaccugucaccaauacuacgguccgcaugaggaccgucugcacuauagacggacuguauauauauauacacauaaaugacaugguaagaua -3'   obs
    cuuuuauauggugugccaaagccauucuuaacacauauauuuauauauauauauggaaauucuauggugaggaugguccguauaagaaccgcaguauuagugaugguuuuucauaguauuaacgaugguuuuuuagaaaaucgucaccaauacuauaaaaaccugucaccaauacuacgguccgcaugaggaccgucugcacuauagacggacuguauauauauauacacauaaaugacaugguaagaua        exp
    .......(((((.........)))))(((((.(((((.(((.((((((((((((((((....((((((((((.(((((((((.(((..(.((((.((((((.((((((((((((.((((((((...(((((((((....)))))))))...))))))))))))))).)))))).))))))).)))).)..))).))))))))).)))))))))....))))))))))))))))...))).)))...)).)))))..
```

read	reads	mm	sample
uauauauauauGuggaaauucuau	1	1	LT-2
auauauauauGuggaaauucuaug	2	1	JCK-2
auauauauauGuggaaauucuaug	2	1	JCK-3
auauauauauGuggaaauucuaug	1	1	JCK-1
uauauauGuggaaauucuaug	1	1	JCK-3
uauauauauggaaauucuGug	1	1	LT-1
auauauGuggaaauucuaug	1	1	LT-1
auauauGuggaaauucuaugg	1	1	LT-2
auauauauggaaauucuaugg	1	0	LT-1
auauauGuggaaauucuaugguga	1	1	JCK-2
uauauGuggaaauucuauggu	1	1	JCK-1
uauauauggaaauucuaugCugag	1	1	JCK-2
auauGuggaaauucuauggu	1	1	LT-3
auauauggaaauuGuauggugag	1	1	LT-2
auauauggaaauucuauggugag	1	0	JCK-3
auauauggaaauucuauggugag	1	0	JCK-2
auGuauggaaauucuauggugag	7	1	LCK-3
auauauggaaauucuauggugagg	2	0	JCK-3
auauauggaaauucuauggugagg	3	0	LT-1
auauauggaaauuGuauggugagg	1	1	LT-1
auauGuggaaauucuauggugagg	6	1	JCK-3
auauauggaaaCucuauggugagg	1	1	LT-3
auauauggaaauucuauggugagg	1	0	LT-3
auauauggaaauucuaugAugagg	2	1	JCK-2
auauauggaaaAucuauggugagg	1	1	LT-3
auauauggaaauucuaugguUagg	1	1	LT-2
auauGuggaaauucuauggugagg	3	1	JCK-1
auauGuggaaauucuauggugagg	11	1	JCK-2
auauGuggaaauucuauggugagg	1	1	LT-2
auauauggaaauucuaugAugagg	1	1	JCK-1
auauaugUaaauucuauggugagg	1	1	LT-1
auauauggaaauucuaugAugagg	3	1	JCK-3
auauauggaaauucuauggugagg	1	0	JCK-2

Star | Mature

cuuuuauauggugugccaaagccauucuuaacacauauauuuauauauauauauggaaauucuauggugaggaugguccguauaagaaccgcaguauuagugaugguuuuucauaguauuaacgaugguuuuuagaaaaucgucaccaauacuauaaaaaccugucaccaauacuacgguccgcaugaggaccgucugcacuauagacggacuguauauauauauacacauaaaugacaugguaagaua

uauGuggaaauucuauggugagga	1	1	JCK-2
auauggaaauucuauggGg	1	1	JCK-2
auauggaaauucuauggug	1	0	LCK-3
auauggaaauucuauCguga	2	1	LT-1
auauggCaauucuauggugag	1	1	LT-2
auauggaaauucuaugCugag	1	1	LT-3
auauggaaauucuauggCgag	2	1	LT-3
auauggaaauucuauggugag	7	0	LT-2
auGuggaaauucuauggugagg	1	1	LT-1
auauggaaauucuaugguUagg	1	1	JCK-2
auauggaaauucuaugguUagg	2	1	JCK-3
auauggaaauucuauggugagg	1	0	JCK-1
auauggaaauucuauggugagga	1	0	LT-3
auauggaaauucuauggugaggau	1	0	JCK-3
uauggaaauucuauggug	1	0	LT-2
uauggaUauucuauggugagg	2	1	JCK-2
uUuggaaauucuauggugagg	1	1	JT-2
uauggaaauucuauggugagga	1	0	LT-3
uauggaaauucuaugAugagga	4	1	LCK-3
auggaaauuGuaugguga	4	1	LT-2
Guggaaauucuauggugag	2	1	JCK-2
auggaaauucuauggugag	2	0	LT-2
auggaaauucuaugCugag	1	1	JCK-3
auggaaauucuaugCugag	1	1	JCK-1
Guggaaauucuauggugag	1	1	JCK-3
auggaaauuGuauggugagg	1	1	LT-2
auggaaauucuauggugagg	4	0	LT-3
auggaaauucuauggugagg	2	0	LT-1
auggaaauucuauggugagg	4	0	JCK-2
auggaaauuGuauggugagg	6	1	LCK-3
auggaaauucuauggugagg	6	0	JT-2
auggaaauucuauggugagg	4	0	LT-2
auggaaauucuauggugagg	1	0	JCK-3
auggaaauucuauggugagg	4	0	JCK-1
auggaaauuGuauggugagg	2	1	JCK-3
auggaaauucuauggugagg	2	0	JT-3
auggaaauucuaugCugagg	1	1	JCK-2
auggaaauuGuauggugagg	3	1	JCK-1
auggaaauuGuauggugagg	5	1	JCK-2
auggaaauuGuauggugagga	1	1	LT-3
auggaaauucuauggugaggaug	1	0	LT-3
auggaaauucuauggugaggaugg	1	0	LT-3
auggaaauucuauggugaggaugg	3	0	JCK-2
auggaaauucuauggugaggaugg	2	0	JCK-3
auggaaauucuauggugaggaugg	3	0	JCK-1
auggaaauucuauggugaggaAgg	4	1	JCK-3
auggaaauuGuauggugaggaugg	1	1	LT-3
auggaaauucuauggugaggaAgg	3	1	JCK-2
auggaaauucuauggugaggauggu	1	0	LT-1
auggaaauucuauggugaggauggA	2	1	JCK-2
uggaaauuGuauggugaggau	1	1	LT-3
uggaaauuGuauggugaggaug	1	1	LT-1
uggaaauuGuauggugaggaugg	3	1	LT-1
uggaaauucuauggugaggaugg	1	0	LT-1
gaaauucuauggugaggau	1	0	LT-3
aauucuauggugaggauggu	1	0	LT-1
aauucuauggugGggaugguccg	1	1	LT-1
aauucuauggugaggaugguUcgu	1	1	JCK-2
auucuauggugaggaAgguccg	1	1	JCK-1
auucuauggugaggauAguccgu	1	1	LT-3
auucuauggugaggaugguccAu	2	1	LT-1
auucuauggugagCaugguccgu	2	1	LT-2
uucuauggugaggaugguc	1	0	LT-3
ucuauggugaggaugguccc	1	0	JCK-2
auggugaggaugguccgua	2	0	LT-2
Cuggugaggaugguccgua	1	1	LT-2
ggugaggaugguccgCauaagaac	3	1	LT-3
gaggaCgguccguauaaga	1	1	LT-3
gaggaugguccguauaagaac	2	0	LT-3
gaggaugguccgCauaagaac	1	1	LT-1

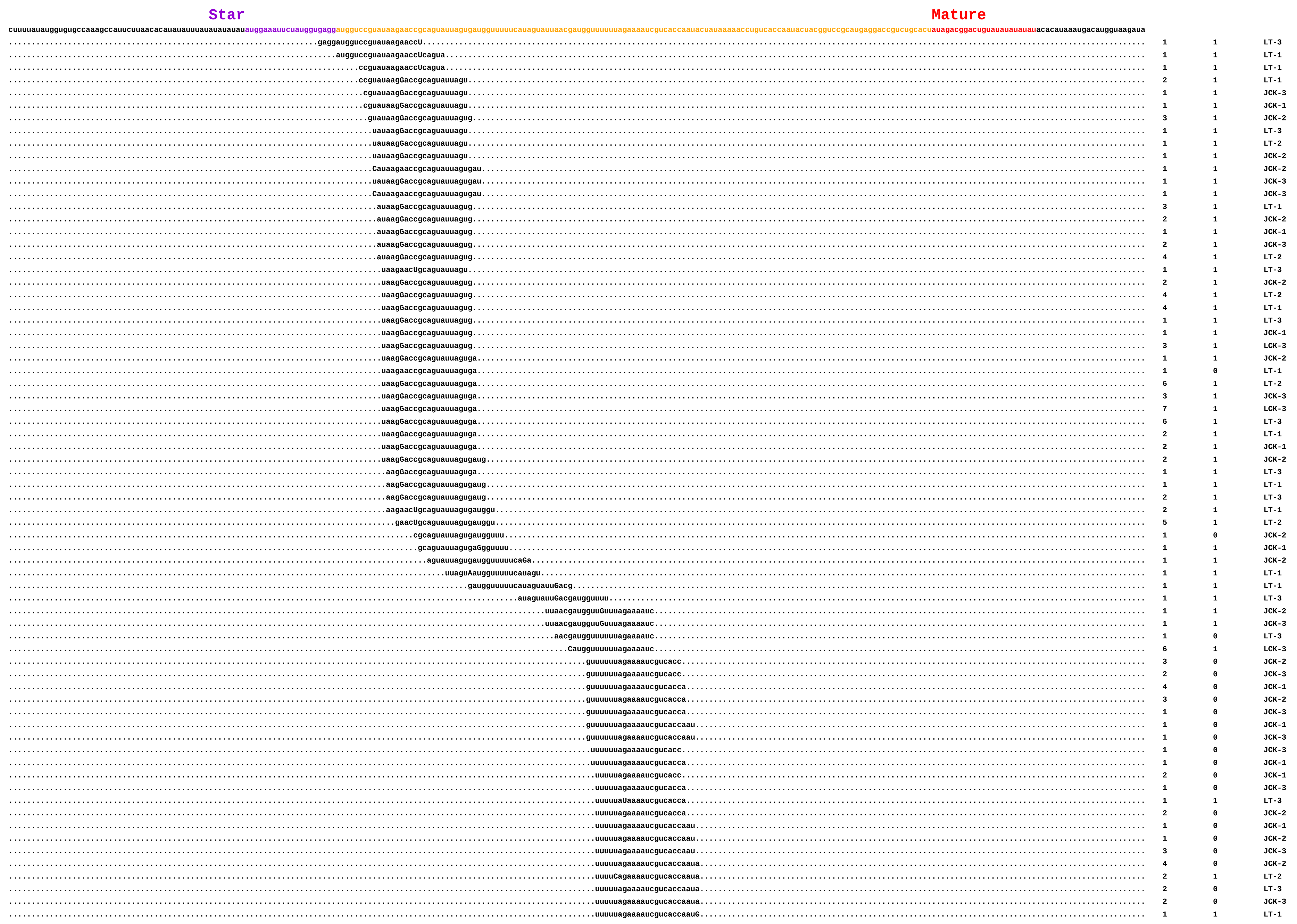
Star
Mature

Star — **Mature**

cuuuuauauggugugccaaagccauucuuaacacauauauuuauauauauauauggaaauucuauggugaggaugguccguauaagaaccgcaguauuagugaugguuuuucauaguauuaacgaugguuuuuuagaaaaucgucaccaauacuauaaaaaccugucaccaauacuacgguccgcaugaggaccgucugcacuauagacggacuguauauauauauacacauaaaugacaugguaagaua

uuuuuagaaaaucgucaccaaua	4	0	LT-1
uuuuuagaaaaucgucaccaauG	1	1	JCK-3
uuuuuagaaaaucgucaccaaua	3	0	JCK-1
uuuuuagaaaaucgucaccaaua	2	0	LT-2
uuuuagaaaaucgucaccaa	4	0	JT-2
uuuuagaaaaucgucaccaa	3	0	JT-3
uuuuagaaaaucgucaccaau	1	0	JCK-3
uuuuagaaaaucgucaccaau	1	0	JCK-2
uuuuagaaaaucgucaccaaua	1	0	JCK-2
uuuuagaaaaucgucaccaaua	1	0	JCK-1
uuuuagaaaaucgucaccaauaAu	2	1	JCK-1
uuuuagaaaaucgucaccaauGcu	1	1	JCK-1
uuuuagaaaaucgucaccaauacu	12	0	JCK-3
uuuuagaaaaucgucaccaauacu	6	0	JCK-2
uuuuagaaaaucgucaccaauacu	2	0	JCK-1
uuCagaaaaucgucacca	1	1	JCK-3
uuuagaaaaucgucacca	1	0	JCK-2
uuuagaaaaucgucaccaau	10	0	LCK-3
uuuagaaaaucgucaccaaua	1	0	LT-2
uuuagaaaaucgucaccaauU	1	1	LT-3
uuuagaaaaucgucaccaauac	1	0	LT-3
uuuagaaaaucgucaccaauacua	1	0	JCK-3
uuuagaaaaucgucaccaauacua	1	0	LT-1
uuuagaaaaucgucaccaauacua	2	0	LT-3
uuuagaaaaucgucaccaauacua	5	0	JCK-2
uuagaaaaucgCcaccaau	5	1	LT-2
uuagaaaaucgucaccaau	3	0	JCK-2
uuagaaaaucgucaccaau	7	0	LT-1
uuagaaaaucgucaccaau	9	0	LCK-3
uuagaaaaucgucaccaau	5	0	LT-2
uuagaaaaucgucaccaauG	2	1	JCK-3
uuagaaaaucgucaccaaua	7	0	LT-1
uuagCaaaucgucaccaaua	3	1	JCK-3
uuagaaaaucgucaccaGua	1	1	JCK-1
uuagaaaaucgucaccaaua	10	0	LT-2
uuagaaaaucgucaccaaua	11	0	LT-3
uuagaaaaucgucaccaauU	3	1	JT-2
uuagaaaaucgucaccaaua	9	0	JCK-2
uuagaaaaucgucaccaauU	11	1	LT-2
uuagaaaaucgucaccaaua	5	0	LCK-3
uuagaaaaucgucaccaaua	9	0	JCK-3
uuagaaaaucgucaccaauU	10	1	JT-3
uuagaaaaucgucaccaaua	4	0	JCK-1
uuagaaaaucgucaccaauU	1	1	JCK-2
uuCgaaaaucgucaccaaua	1	1	LT-1
uuagaaCaucgucaccaaua	2	1	LT-3
uuagaaaaucgucaccaauU	1	1	LT-3
uuagaaaaucgucaccaauac	1	0	JCK-2
uuagaaaaucgucaccaauac	1	0	JCK-3
uuagaaaaucgucaccaauac	3	0	LT-3
Cuagaaaaucgucaccaauac	1	1	LT-2
uuagaaUaucgucaccaauacu	1	1	LT-3
uuagaaaaucgucaccaauacu	1	0	JCK-3
uuagaaaaucgucaccaauacu	3	0	LT-2
uuagaaaaucgucaccaauacu	3	0	JCK-2
uuagaaaaucgucaccaauacu	1	0	JCK-1
uuagaaaaucgucaccaauacu	8	0	LT-3
uuagaaaaucgucaccaauacu	4	0	LCK-3
uuagaaaaucgucaccaGuacu	7	1	LCK-3
uuagaaaaucgucaGcaauacu	3	1	JCK-3
uuaUaaaaucgucaccaauacua	2	1	JCK-2
uuagaaaaucgucaccaauacua	11	0	JCK-1
uuaUaaaaucgucaccaauacua	1	1	JCK-1
uuaUaaaaucgucaccaauacua	2	1	JCK-3
uuagaaaaucgucaccaGuacua	1	1	JCK-1
uuagaaaaucgucaccaauacua	10	0	JCK-2
uuagaaaaucgucaccaGuacua	2	1	JCK-2
uuagaaaaucgucaccaGuacua	3	1	JCK-3
uuagaaaaucgucaccaauacuU	2	1	JCK-3
uuaUaaaaucgucaccaauacua	1	1	LT-1

Star — Mature

cuuuuauauggugugccaaagccauucuuaacacauauauuuauauauauauauggaaauucuauggugaggaugguccguauaagaaccgcaguauuagugaugguuuuucauaguauuaacgaugguuuuuuagaaaaucgucaccaauacuauaaaaaccugucaccaauacuacgguccgcaugaggaccgucugcacuauagacggacuguauauauauauacacauaaaugacaugguaagaua

uuagaaaaucgucaccaauacuU	1	1	JCK-1
uuagaaaaucgucaccaauacua	3	0	LCK-3
uuagaaaaucgucaccaauacua	1	0	LT-3
uuagaaaaucgucaccaauacua	3	0	LT-1
uuagaaaaucgucaccaauacua	8	0	JCK-3
uuagaaaaucgucaccaauacuUu	4	1	LCK-3
uuagaaaaucgucaccaauacuau	2	0	LT-3
uuagaaaaucgucaccaauacuau	7	0	JCK-3
uuagaaaaucgucaccaauacuUu	2	1	LT-3
uuagaaaaucgucaccaauacuau	1	0	LT-2
uuagaaaaucgucaccaauacuau	9	0	LT-1
uuaUaaaaucgucaccaauacuau	1	1	JCK-2
uuagaaaaucgucaccaauacuUu	1	1	LT-1
uuagaaaaucgucaccaaGacuau	1	1	JCK-3
uuagaaaaucgucaccaauacuUu	1	1	LT-2
uuagaaaaucgucaccaauacuau	5	0	JCK-1
uuagaaaaucgucaccaauacuau	14	0	JCK-2
uagaaaaucgucaccaaua	2	0	LT-1
uagaaaaucgucaccaauU	1	1	JCK-1
uagaaaaucgucaccaaua	1	0	LT-3
uagaaaaucgucaccaauU	1	1	JCK-3
uagaaaaucgucaccaauU	2	1	JCK-2
uagaaaaucgucaccaauUc	1	1	LT-2
uagaaaaucgucaccaauacu	1	0	LT-2
uagaaaaucgucaccaauacuau	1	0	JCK-1
uagaaaaucgucaccaauacuau	2	0	LT-3
uagaaaaucgucaccaauacuaua	1	0	LCK-3
uaAaaaaucgucaccaauacuaua	1	1	JCK-1
uagaaaaucgucaccaauacuaua	1	0	JCK-1
uaUaaaaucgucaccaauacuaua	1	1	LT-2
uagaaaaucgucaccaauacuaua	3	0	LT-2
uagaaaaucgucaccaauacuaua	1	0	JCK-2
uaAaaaaucgucaccaauacuaua	1	1	JCK-3
agaaaaucgucaccaaua	15	0	JT-3
agaaaaucgucaccaaua	10	0	JT-2
agaaaaucgucaccaaua	4	0	LT-3
agaaaaucgucaccaaua	11	0	LCK-3
agaaaaucgucaccaaua	4	0	JCK-1
agaaaaucgucaccaaua	6	0	JCK-2
agaaaaucgucaccaaua	1	0	LT-1
agaaaaucgucaccaaua	7	0	JCK-3
agaaaaucgucaccaauU	1	1	LT-2
agaaaaucgucaccaaua	7	0	LT-2
agCaaaucgucaccaauacu	1	1	JCK-2
agaaaaucgucaccaauacu	7	0	JCK-2
agaaaaucgucaccaauacu	22	0	LT-2
agaaaaucgucaccaauacu	1	0	JT-3
agaaaaucgucaccaauacu	5	0	JCK-3
agaaaaucgucaccaauacu	13	0	LT-1
agaaaCucgucaccaauacu	1	1	LT-2
agaaaaucgucaccaauacu	9	0	LCK-3
agaaaaucgucaccaauacu	17	0	LT-3
agaaaaucgucaccaGuacu	1	1	LT-3
agaaaaucgucaccaaAacu	1	1	LT-1
agaaaaucgucaccaauacu	1	0	JCK-1
agaaaaucgucaccaauacu	5	0	JT-2
agaaaaucgucaccaauacua	11	0	JCK-3
agaaaaucgucaccaauacuC	1	1	LT-2
agaaaaucgucaccaauacua	11	0	JCK-1
agaaaaucgucaccaauacua	10	0	JCK-2
agaaaaucgucaccaauacua	3	0	LT-2
agaaaaucguGaccaauacua	1	1	JCK-1
agaaaaucgucaccaauacua	4	0	JT-3
agaaaaucgucaccaauacuU	1	1	LT-1
agaaaaucgucaccaauacua	2	0	LT-3
agaaaaucgucaccaauacua	2	0	JT-2
agaaaaucgucaccaauacuG	1	1	LT-1
agaaaaucgucaccaauacua	1	0	LT-1
agaaaaucgucaccaaAacuau	1	1	LT-1
agaaaaucgucaccaauacuau	3	0	LT-2

Star **Mature**

cuuuuauauggugugccaaagccauucuuaacacauauauuuauauauauauauggaaauucuauggugaggaugguccguauaagaaccgcaguauuagugaugguuuuucauaguauuaacgaugguuuuuuagaaaaucgucaccaauacuauaaaaaccugucaccaauacuacgguccgcaugaggaccgucugcacuauagacggacuguauauauauauacacauaaaugacaugguaagaua

agaaaaucgucaccaauacuau	4	0	JCK-3
agaaaaucgucaccaauacuau	2	0	JCK-2
agaaaaucgucaccaauacuau	3	0	LT-3
agaaaaucgucaccaauacuaua	31	0	JCK-3
agaaaaucgucaccaauacuaua	10	0	LT-2
agCaaaucgucaccaauacuaua	1	1	JCK-3
agaaaaucgucaccaauacuaua	6	0	LT-1
agaaaaucgucaccaCuacuaua	5	1	JCK-3
agaaaaucgucaccaauacuaCa	1	1	LT-1
agaaaaucgucaccaauacCaua	1	1	LT-1
agaaaaucgCcaccaauacuaua	2	1	JCK-1
aAaaaaucgucaccaauacuaua	1	1	JCK-1
agaaaaucgucaccaauacuaNa	1	1	JCK-1
aUaaaaucgucaccaauacuaua	1	1	JCK-3
aUaaaaucgucaccaauacuaua	2	1	LT-2
agaaaGucgucaccaauacuaua	1	1	JCK-2
agaaaaucgucaccaauacuaua	20	0	JCK-1
agaaaaucgucaccaauacuaCa	1	1	LT-3
agaaaCucgucaccaauacuaua	1	1	LT-1
aAaaaaucgucaccaauacuaua	2	1	JT-3
agaaaaucgucaccaauacuaua	1	0	JT-3
agaaaaucgucaccaauacuaua	4	0	LT-3
aUaaaaucgucaccaauacuaua	2	1	JCK-2
aAaaaaucgucaccaauacuaua	2	1	JCK-2
aUaaaaucgucaccaauacuaua	1	1	JCK-1
agaaaaucgucaccaauacuaua	1	0	JT-1
Ggaaaaucgucaccaauacuaua	1	1	LT-1
agaaaaucgucaccaauacuaua	52	0	JCK-2
agaaaaucgucaccaauacuauaa	1	0	LT-1
agaaaaucgucaccaauacuauaa	2	0	JCK-2
gaaaaucgucaccaauacua	1	0	LT-1
Caaaaucgucaccaauacuau	1	1	LT-3
gaaaaucgucaccaauacuaua	3	0	JCK-1
Aaaaaucgucaccaauacuaua	1	1	JCK-3
gaaaaucgucaccaauacuauaa	1	0	LT-3
aaaaucgucaccaauacuaua	2	0	JCK-2
aaaaucgucaccaauacuauaaa	1	0	LT-3
Aucaccaauacuauaaaaaccugu	1	1	JCK-1
Aucaccaauacuauaaaaaccugu	3	1	JCK-2
Aucaccaauacuauaaaaaccugu	1	1	JCK-3
ucaccaauacuauaaaaaGcuguc	1	1	JCK-1
ucaccaauacuauaaaaaGcuguc	2	1	JCK-2
ucaccaauacuauaaaaaGcuguc	2	1	LT-1
caauacuauaaaaaccuguc	1	0	JCK-2
ccaauacuGcgguccgcauga	2	1	JCK-3
gguccgcaugagAaccgucugc	1	1	LT-3
uccgcaugagAaccgucug	3	1	JCK-1
uccgcaugagAaccgucug	1	1	JCK-3
uccgcaugaggaUcgucugc	1	1	JCK-2
uccgcaugagAaccgucugc	2	1	LT-2
uccgcaugCggaccgucugc	1	1	JCK-1
uccgcaugaAgaccgucugca	1	1	LT-3
uccgcaugaggaccgucugca	1	0	LT-1
uccgcaugaggaccgucugUa	2	1	JCK-2
uccgcaugaAgaccgucugcacu	3	1	LT-3
uccgcaugagAaccgucugcacu	1	1	LT-1
uccgcaugaggaccgucugUacu	2	1	JCK-1
uccgcaugagAaccgucugcacu	2	1	LT-2
ccgcaugagAaccgucugc	1	1	LT-3
ccgcaugaggaccgucugU	3	1	LCK-3
ccgcaugagAaccgucugca	1	1	LT-1
ccgcaugagAaccgucugca	4	1	LT-2
ccgcaugaggaccgucugUa	1	1	LT-1
ccgcaugaggaccgucugcacC	2	1	LT-3
ccgcaugaggaccgucugcacCa	2	1	LT-3
cgcaugaggaccgucugUa	1	1	JCK-2
cgcaugaAgaccgucugcac	1	1	JCK-1
cgcaugaAgaccgucugcac	3	1	JCK-2
cgcaugaggaccgucugcaAu	1	1	JCK-2
caugagAaccgucugcacu	4	1	LT-3

```
                                       Star                                                                                                        Mature
cuuuuauauggugugccaaagccauucuuaacacauauauuuauauauauauauggaaauucuauggugaggaugguccguauaagaaccgcaguauuagugaugguuuuucauaguauuaacgaugguuuuuuagaaaaucgucaccaauacuauaaaaaccugucaccaauacuacgguccgcaugaggaccgucugcacuauagacggacuguauauauauauacacauaaaugacaugguaagaua
........................................................................................................................................................................................caugagAaccgucugcacu...............................................  1  1  LT-2
.........................................................................................................................................................................................augaggaccAucugcacu...............................................  1  1  LT-2
.........................................................................................................................................................................................augagAaccgucugcacuauagac.........................................  1  1  JCK-2
.........................................................................................................................................................................................augagAaccgucugcacuauagac.........................................  1  1  LT-3
.........................................................................................................................................................................................augagAaccgucugcacuauagac.........................................  2  1  JCK-3
..........................................................................................................................................................................................ugaggaccgucugUacuaua............................................  1  1  LT-3
..........................................................................................................................................................................................ugaggaccgucugcacuaua............................................  1  0  JCK-3
..........................................................................................................................................................................................ugaggaccgucugcacuaua............................................  2  0  JCK-2
..........................................................................................................................................................................................ugaggaccAucugcacuauagac.........................................  1  1  LT-1
...........................................................................................................................................................................................gagAaccgucugcacuauagac.........................................  1  1  LT-2
...........................................................................................................................................................................................gagAaccgucugcacuauagac.........................................  2  1  LT-3
...........................................................................................................................................................................................gagAaccgucugcacuauagac.........................................  1  1  LT-1
...........................................................................................................................................................................................gaggaccAucugcacuauagacgg.......................................  2  1  LT-3
...........................................................................................................................................................................................gaggaccAucugcacuauagacgg.......................................  3  1  LT-1
............................................................................................................................................................................................aggaccAucugcacuauagacgga......................................  1  1  LT-3
............................................................................................................................................................................................aggaccAucugcacuauagacgga......................................  1  1  LT-1
...................................................................................................................................................................................................ucugcacuauagacgAacu....................................  1  1  LT-2
...................................................................................................................................................................................................ucugcacuauagacgAacugu..................................  3  1  LT-2
....................................................................................................................................................................................................cugcacuauagacggacuCu..................................  2  1  LT-3
....................................................................................................................................................................................................cugcacuauagacggacuAu..................................  1  1  LT-3
....................................................................................................................................................................................................cugcacuauagacggacuCua.................................  4  1  LT-2
....................................................................................................................................................................................................cugcacuauagacgAacuguaua...............................  2  1  JCK-2
....................................................................................................................................................................................................cugcacuauagacgAacuguaua...............................  2  1  JCK-3
....................................................................................................................................................................................................cugcacuauagacgAacuguauau..............................  1  1  JCK-1
....................................................................................................................................................................................................cugcacuauagacgAacuguauau..............................  1  1  JCK-2
....................................................................................................................................................................................................cugcacuauagacggacuguauauU.............................  1  1  LT-2
.....................................................................................................................................................................................................ugcacuauagacggacuC...................................  2  1  JCK-2
.....................................................................................................................................................................................................ugcacuauagacggacuAua.................................  1  1  LT-1
.....................................................................................................................................................................................................ugcacuauagacggacuCua.................................  1  1  LT-1
......................................................................................................................................................................................................gcacuauaAacggacuguau................................  1  1  JCK-1
......................................................................................................................................................................................................gcacuauagacggacuguauaua.............................  1  0  LT-1
.......................................................................................................................................................................................................cacuauagacgAacuguaua...............................  4  1  LT-2
.......................................................................................................................................................................................................cacuauagacgAacuguauau..............................  5  1  LCK-3
........................................................................................................................................................................................................acuauagacggacuguaCa...............................  1  1  LT-3
........................................................................................................................................................................................................acuauagacggacuguauau..............................  2  0  LT-3
........................................................................................................................................................................................................acuauagacggacuguauaua.............................  1  0  JCK-3
........................................................................................................................................................................................................acuauagacggacuguauauau............................  1  0  LT-1
........................................................................................................................................................................................................acuauagacggacuguauauaCa...........................  1  1  JCK-1
........................................................................................................................................................................................................acuauagacggacuguauauaua...........................  1  0  JCK-2
.........................................................................................................................................................................................................cuauagacggacuguauU...............................  1  1  JCK-3
.........................................................................................................................................................................................................cuauagacggacugAaua...............................  1  1  JCK-2
.........................................................................................................................................................................................................cuauagacggacuguauC...............................  1  1  LT-2
.........................................................................................................................................................................................................cuauaAacggacuguaua...............................  2  1  JT-2
.........................................................................................................................................................................................................cuauagacggacuguaua...............................  2  0  LT-3
.........................................................................................................................................................................................................cuauagacggacuguauU...............................  1  1  LT-1
.........................................................................................................................................................................................................cuauaAacggacuguaua...............................  1  1  JT-3
.........................................................................................................................................................................................................cuauagacggaUuguaua...............................  2  1  LT-3
.........................................................................................................................................................................................................cuauagacggacuguauau..............................  1  0  JCK-3
.........................................................................................................................................................................................................cuauagacggacuguauau..............................  1  0  LT-3
.........................................................................................................................................................................................................cuauagacAgacuguauau..............................  1  1  LT-3
.........................................................................................................................................................................................................cuauagacggacuguauaA..............................  3  1  LT-2
.........................................................................................................................................................................................................cuauagacggacuguauUu..............................  2  1  LT-1
.........................................................................................................................................................................................................cuauagacggacuguauau..............................  3  0  JCK-2
.........................................................................................................................................................................................................cuauagacggacuguauUu..............................  2  1  LT-3
.........................................................................................................................................................................................................cuauagacggacuguGuaua.............................  2  1  JCK-2
.........................................................................................................................................................................................................cuauagacggacugAauaua.............................  2  1  LT-1
.........................................................................................................................................................................................................cuauagacggacuguGuaua.............................  3  1  JCK-3
.........................................................................................................................................................................................................cuauagacggacuguauauC.............................  3  1  JCK-2
.........................................................................................................................................................................................................cuauagacggacuguauauU.............................  1  1  LT-1
.........................................................................................................................................................................................................cuauagacggacuguauauU.............................  5  1  LT-3
.........................................................................................................................................................................................................cuauagacggacuguauauC.............................  1  1  JCK-1
.........................................................................................................................................................................................................cuauagacggacuguauUua.............................  1  1  JCK-2
.........................................................................................................................................................................................................cuauagacggacuguauauU.............................  1  1  JCK-2
.........................................................................................................................................................................................................cuauagacggacuguauauC.............................  2  1  JCK-3
.........................................................................................................................................................................................................cuauagacggacuguauauCu............................  1  1  JCK-2
.........................................................................................................................................................................................................cuauagacggacuguauauUu............................  3  1  JCK-1
.........................................................................................................................................................................................................cuauagacggacuguauauUu............................  2  1  LT-1
.........................................................................................................................................................................................................cuauagacggacuguauauCu............................  1  1  JCK-1
.........................................................................................................................................................................................................cuauagacggacuguauauUu............................  2  1  LT-3
.........................................................................................................................................................................................................cuauagacggacuAuauauau............................  1  1  LT-2
```

Star   Mature

cuuuuauauggugugccaaagccauucuuaacacauauauuuauauauauauauggaaauucuauggugaggauggccguauaagaaccgcaguauuagugaugguuuuucauaguauuaacgaugguuuuuuagaaaaucgucaccaauacuauaaaaaccugucaccaauacuacgguccgcaugaggaccgucugcacuauagacggacuguauauauauauacacauaaaugacaugguaagaua

cuauagacggacuguauauUu	6	1	JCK-2
cuauagacggacuguauauUu	3	1	LT-2
cuauagacggacuguauauaA	1	1	LT-3
cuauagacggacuAuauauauau	1	1	JCK-3
uauagacggacuguauGua	2	1	LT-2
uauagacggacuguauauUu	2	1	LT-3
uauagacggacuguaCauaua	1	1	LT-3
uauaAacggacuguauauaua	1	1	JCK-1
uauaAacggacuguauauaua	1	1	JCK-3
uauagacggacuguauGuauau	1	1	LT-2
uauagacggacuguauauauau	1	0	JCK-3
uauagacggacuguauauauau	1	0	JCK-1
uauagacggacuguauGuauau	1	1	LT-1
uauagacggacuAuauauauau	1	1	JCK-1
uauagacAgacuguauauauau	1	1	LT-3
uauagacggacuguauauauaCa	1	1	LT-1
uauagacggaUuguauauauaua	1	1	LT-3
uauagacggacuguGuauauaua	4	1	JCK-2
uauaAacggacuguauauauaua	1	1	JCK-1
uauagacggacuguauaCauaua	2	1	JCK-2
uauaAacggacuguauauauaua	2	1	JCK-3
uauagacggacuAuauauauauau	2	1	LT-2
uauagacggacuAuauauauauau	1	1	LT-1
uauagacggacuAuauauauauau	3	1	JCK-1
uauagacggacuAuauauauauau	6	1	JCK-2
uauagacggacuAuauauauauau	5	1	JCK-3
auagacggacuguauaua	1	0	LT-1
auagacggacuguauaua	1	0	LCK-3
auagacggacuguauGua	3	1	LT-3
auagacggaUuguauaua	1	1	LT-2
auagacggacuguauauC	2	1	LT-1
auagacggacuguauauUu	1	1	LT-3
auagacggacuguauauUu	2	1	LT-2
auagacggacuguauauau	2	0	LT-2
auagacggacuguauauau	1	0	LT-3
auagacggacuguauauau	1	0	JCK-2
auagacggacuguauauauC	2	1	LT-3
auagacggacuguauauauU	1	1	LT-3
auagacggacuguauauauC	1	1	JCK-3
auagacggacuguauauauC	1	1	JCK-1
auagacggacuguauauaua	1	0	JCK-2
auagacggacuguauauaua	1	0	JCK-3
auagacggacuguauauauC	2	1	JCK-2
auagacggacuguauauauau	3	0	JCK-1
auagacggacuguauauauau	3	0	JCK-2
auagacggacuguauauauau	1	0	LT-2
auagacggacuguauauauau	2	0	LT-3
auagacgAacuguauauauau	1	1	LT-1
auagacggacuguauGuauau	1	1	LT-3
auagacggacuguauauauaua	2	0	JCK-1
auagacggacuguauauauaua	1	0	LT-1
auagacggacuguauauauauC	1	1	JCK-1
auagacggacugGauauauaua	1	1	JCK-2
auagacggacuguauGuauaua	5	1	LCK-3
auagacggacuguaCauauaua	1	1	LT-1
auagacggacuguauauauauC	5	1	JCK-2
auagacggacuguauauauaua	7	0	JCK-2
auagacggacuguauauauauC	1	1	JCK-3
auagacggacuguauauauauC	3	1	LT-2
auagacggacuguauauauaua	3	0	JCK-3
auagacggacuguauUuauaua	1	1	JCK-3
auagacggacuguauauaGauau	1	1	LT-1
auagacgAacuguauauauauau	1	1	LT-3
auagacggacuguauauauauau	26	0	JCK-1
auagacggaUuguauauauauau	1	1	JCK-3
auagacggacuguauGuauauau	1	1	JCK-3
auagacggacuguauauauauau	53	0	JCK-2
auagacggacuguauauauauau	1	0	LT-2
auagacggacuguauauaCauau	7	1	JCK-2
auagacggacuguauauauauau	33	0	JCK-3

Star | Mature

cuuuuauauggugugccaaagccauucuuaacacauauauuuauauauauauauggaaauucuauggugaggaugguccguauaagaaccgcaguauuagugaugguuuuucauaguauuaacgaugguuuuuuagaaaaucgucaccaauacuauaaaaaccugucaccaauacuacgguccgcaugaggaccgucugcacuauagacggacuguauauauauauacacauaaaugacaugguaagaua			
...auagacgAacuguauauauauau...	4	1	JCK-1
...auagacgAacuguauauauauau...	2	1	JCK-2
...auagacggacuguauauauauau...	1	0	LCK-3
...auagacggacuguauauauauau...	2	0	LT-1
...auagacggacuguauaCauauau...	1	1	JCK-2
...auagacgAacuguauauauauau...	1	1	LT-1
...auagacgAacuguauauauauau...	1	1	LT-2
...auagacggacuguauauauCuau...	1	1	LT-1
...auagacggacuguauauaCauau...	2	1	JCK-3
...auagacggacuguauGuauauau...	4	1	JCK-2
...auagacggacuguauauaCauau...	1	1	JCK-1
...auagacggacuguauauauGuau...	1	1	JCK-3
...auagacggacuguauauauauaua...	2	0	LT-1
...auagacggacuguauaCauauaua...	1	1	LT-1
...auagacgAacuguauauauauaua...	2	1	LT-1
...auagacggaUuguauauauauaua...	1	1	JCK-3
...auagacggaUuguauauauauaua...	1	1	JCK-1
...auagacggacuguauauauaGaua...	1	1	JCK-3
...auagacggaAuguauauauauaua...	1	1	JCK-3
...auagacggacuguauauauGuaua...	1	1	JCK-1
...auagacggacuguauauauCuaua...	1	1	LT-1
...auagacgAacuguauauauauaua...	1	1	JCK-1
...auagacggacuguauaCauauaua...	1	1	JCK-2
...auagacggacuguauauauauaua...	22	0	JCK-1
...auagacggacuguauauUuauaua...	1	1	JCK-2
...auagacggacuguauauauUuaua...	1	1	LT-3
...auagacgAacuguauauauauaua...	2	1	LCK-3
...auUgacggacuguauauauauaua...	1	1	JCK-3
...auagacggacuguauauauauaua...	34	0	JCK-2
...auagacggaUuguauauauauaua...	1	1	LT-3
...auagacggacuguauauauauaua...	11	0	JCK-3
...auagacggacuguauauauauauaca...	1	0	LT-1
....uagacggacugGauauau...	1	1	LT-1
....uagacggacuguauauauC...	2	1	LCK-3
....uagacggacuguauauauC...	3	1	LT-2
....uagacggacuguauauauaua...	1	0	JCK-1
....uagacggacuguauauauauC...	1	1	JCK-1
....uagacggacuguauauauaua...	2	0	LT-3
....uagacggacuguauauauaua...	2	0	JCK-3
....uagacggacuguauauaGaua...	1	1	LT-3
....Cagacggacuguauauauaua...	1	1	JCK-3
....uagacggacuguauauauaua...	1	0	JCK-2
....uagacggacuguauauauauau...	13	0	LCK-2
....uagacggacuguauauauauau...	1	0	LCK-3
....uagacggacuguauauauauau...	12	0	LCK-1
....uagacggacuguauGuauauau...	1	1	LT-3
....Cagacggacuguauauauauau...	4	1	JCK-2
....uagacggacuguauauauauCu...	1	1	LT-3
....uagacggacuguauauaGauau...	1	1	JCK-2
....uagacggacuguauauauauau...	40	0	JCK-3
....uagacggacuguauauauauau...	1	0	LT-1
....uagacggacuguauauauauau...	2	0	LT-2
....Cagacggacuguauauauauau...	1	1	JCK-3
....uagacggacuguauauauauau...	3	0	LT-3
....Cagacggacuguauauauauau...	1	1	JCK-1
....uagacggacuguauGuauauau...	2	1	JCK-3
....uagacgAacuguauauauauau...	1	1	JCK-3
....uagacgAacuguauauauauau...	6	1	JCK-1
....uagacggacuguauauUuauau...	1	1	JCK-2
....uagacggacuguauGuauauau...	1	1	JCK-2
....uagacgAacuguauauauauau...	8	1	JCK-2
....uagacggacuguaCauauauau...	1	1	LT-3
....uagacgAacuguauauauauau...	4	1	LT-1
....uagacgAacuguauauauauau...	3	1	LT-3
....uagacggacuguauauaGauau...	1	1	LT-2
....uagacggacuguauauauauau...	23	0	JCK-1
....uUgacggacuguauauauauau...	1	1	JCK-1
....uagacggacuguauauCuauau...	2	1	JCK-2
....uagacggacuCuauauauauau...	1	1	JCK-1
....uagacggacuCuauauauauau...	1	1	JCK-3

Star			
Precursor: cuuuuauauggugugccaaagccauucuuaacacauauauuuauauauauau**auggaaauucuauggugagg**augguccguauaagaaccgcaguauuagugaugguuuucauaguauuaacgaugguuuuuuagaaaaucgucaccaauacuauaaaaaccugucaccaauacuacgguccgcaugaggaccgucugcacu**auagacggacuguauauauauau**acacauaaaugacaugguaagaua			

Mature

Sequence	Count	Mismatch	Library
uagacggacuguauauauauau	51	0	JCK-2
uagacggaUuguauauauauaua	1	1	LT-3
uagacgAacuguauauauauaua	3	1	JCK-2
uagacggacuguauauauauaua	1	0	LT-3
uagacggacuguauauauauauU	1	1	JCK-3
uagacggacuguauauauauaua	45	0	JCK-3
uagacggacuguauauauauauU	4	1	JCK-2
uagacggacuguaCauauauaua	1	1	JCK-3
uagacggacuguauauCuauaua	1	1	LT-1
uagacggacuguauauauauaua	49	0	JCK-2
uagacggacuguauGuauauaua	3	1	JCK-2
uUgacggacuguauauauauaua	1	1	JCK-3
uagacggacuguauauauauaua	44	0	JCK-1
uagacggaUuguauauauauaua	3	1	JCK-3
uagacggaUuguauauauauaua	4	1	JCK-1
uagacggacuguauGuauauaua	2	1	JCK-3
uagacggacuguauauaCauaua	2	1	JCK-1
uagacggacuguauauauauaua	3	0	LT-1
uagacggacuguauauauauauU	3	1	JCK-1
uagacggaUuguauauauauaua	5	1	JCK-2
uagacgAacuguauauauauaua	1	1	JCK-1
uagacgAacuguauauauauaua	1	1	LCK-3
uUgacggacuguauauauauaua	1	1	JCK-1
uagGcggacuguauauauauaua	2	1	JCK-3
uagacgAacuguauauauauaua	2	1	JCK-3
uagacggacuUuauauauauaua	1	1	LT-1
uagacggacuguauauauauauac	18	0	JCK-1
uagacgAacuguauauauauauac	2	1	LCK-3
uagacggacuguauauauCuauac	1	1	JCK-2
Cagacggacuguauauauauauac	1	1	JCK-2
uagacggacuguauauauaGauac	2	1	JCK-2
uagacggacuguauauCuauauac	2	1	JCK-3
uagacggacuguauauauGuauac	1	1	JCK-1
uagacgAacuguauauauauauac	2	1	LT-1
uagacggacuguauauauCuauac	1	1	JCK-3
Cagacggacuguauauauauauac	2	1	JCK-1
uUgacggacuguauauauauauac	8	1	JCK-3
uagacgAacuguauauauauauac	2	1	JCK-2
uaAacggacuguauauauauauac	5	1	JCK-1
Cagacggacuguauauauauauac	2	1	JCK-3
uagacggacuguauauauauauac	44	0	JCK-2
uUgacggacuguauauauauauac	6	1	JCK-1
uUgacggacuguauauauauauac	7	1	JCK-2
uagacgAacuguauauauauauac	2	1	JCK-3
uagacggacuguauauCuauauac	1	1	JCK-2
uGgacggacuguauauauauauac	1	1	JCK-2
uaAacggacuguauauauauauac	5	1	JCK-3
uagacggacuguauauCuauauac	6	1	JCK-1
uagacggacuguaCauauauauac	4	1	JCK-2
uagacggacuguauauauauauac	1	0	LT-3
uagacggacuguauauauGuauac	1	1	LT-2
uagacggacuguauauauauauac	36	0	JCK-3
uaAacggacuguauauauauauac	10	1	JCK-2
uagacggacuguauauauGuauac	5	1	JCK-2
uagacggacuguauauauauauaca	1	0	JCK-3
.agacggacuguauauauaua	2	0	LT-2
.agacggaUuguauauauaua	1	1	LT-3
.agacggacuguauauauaua	2	0	JCK-3
.agacggacuguauauauaua	2	0	JCK-2
.agacggacuguauauUuauau	1	1	JCK-1
.agacggacuguauauauauau	14	0	JCK-2
.agacggacuguauCuauauau	1	1	JCK-1
.agacggacuguauauauauau	2	0	LT-1
.agacggacuguauauauauau	5	0	JCK-1
.agacggacuguauauauauau	13	0	JCK-3
.agacggacuguauauauauaua	2	0	LT-3
.agacggacuguauauauauaua	8	0	JCK-3
.agacggacuguauauauGuaua	1	1	LT-3
.agacggacuguauauauauaua	2	0	LT-1
.agacggaGuguauauauauaua	1	1	JCK-2

Star Mature

```
cuuuuauauggugugccaaagccauucuuaacacauauauuuauauauauauauggaaauucuauggugaggaugguccguauaagaaccgcaguauuagugaugguuuuucauaguauuaacgaugguuuuuuagaaaaucgucaccaauacuauaaaaaccugucaccaauacuacgguccgcaugaggaccgucugcacuauagacggacuguauauauauauacacauaaaugacaugguaagaua
.............................................................................................................................................................................................................agacggaUuguauauauauaua.......................   1    1    LT-1
.............................................................................................................................................................................................................agacggacuguauauauauaua.......................   13   0    JCK-2
.............................................................................................................................................................................................................agacggacuguauauauauaua.......................   2    0    JCK-1
.............................................................................................................................................................................................................agacggacuguauauCuauauac......................   1    1    JCK-1
.............................................................................................................................................................................................................agacggacuguauauauauauac......................   2    0    LT-1
.............................................................................................................................................................................................................aAacggacuguauauauauauac......................   1    1    JCK-3
.............................................................................................................................................................................................................agacggacuguauauauauauUc......................   1    1    JCK-2
.............................................................................................................................................................................................................agacggacuguauauauauauac......................   4    0    JCK-2
.............................................................................................................................................................................................................agacggacuguauauauGuauac......................   1    1    LT-3
.............................................................................................................................................................................................................agacggacuguauauauauauUc......................   3    1    JCK-1
.............................................................................................................................................................................................................agacggacuguauauauauauac......................   1    0    JCK-1
.............................................................................................................................................................................................................Ugacggacuguauauauauauac......................   2    1    JCK-2
..............................................................................................................................................................................................................gacggacuguauauaGaua.........................   1    1    LT-3
..............................................................................................................................................................................................................gacggacuguauauauauau........................   2    0    LT-2
..............................................................................................................................................................................................................gacggacuguauauauauau........................   2    0    JCK-3
..............................................................................................................................................................................................................gacgAacuguauauauauau........................   1    1    LT-2
..............................................................................................................................................................................................................gacggacuguauauauauau........................   1    0    JCK-2
..............................................................................................................................................................................................................gacggacuguauauauauau........................   1    0    LT-3
..............................................................................................................................................................................................................gacggacuCuauauauauau........................   1    1    LT-1
..............................................................................................................................................................................................................Cacggacuguauauauauau........................   1    1    JCK-3
..............................................................................................................................................................................................................gacggacuguauauauauau........................   2    0    JCK-1
..............................................................................................................................................................................................................gacggacuguauauauauau........................   2    0    LT-1
..............................................................................................................................................................................................................gacggacuguauauauauaua.......................   12   0    JCK-2
..............................................................................................................................................................................................................gacgAacuguauauauauaua.......................   1    1    LT-2
..............................................................................................................................................................................................................gacgAacuguauauauauaua.......................   1    1    JT-2
..............................................................................................................................................................................................................gacggacuguauauauauaua.......................   1    0    LT-2
..............................................................................................................................................................................................................gacggacuguauauauGuaua.......................   4    1    LT-2
..............................................................................................................................................................................................................gacggacuguauauauauaua.......................   1    0    LT-3
..............................................................................................................................................................................................................gacggacuguauauauauaua.......................   4    0    JT-1
..............................................................................................................................................................................................................gacggaUuguauauauauaua.......................   2    1    LT-2
..............................................................................................................................................................................................................gacggacuguauauauauaua.......................   3    0    LT-1
..............................................................................................................................................................................................................gacgAacuguauauauauaua.......................   1    1    LT-3
..............................................................................................................................................................................................................gacggacuguauauauauaua.......................   4    0    JCK-3
..............................................................................................................................................................................................................gacUgacuguauauauauaua.......................   2    1    JCK-2
..............................................................................................................................................................................................................gacggacuguauauauauaua.......................   2    0    JCK-1
..............................................................................................................................................................................................................gacgAacuguauauauauaua.......................   2    1    JT-3
..............................................................................................................................................................................................................gacggacuguauauCuauaua.......................   2    1    LT-3
..............................................................................................................................................................................................................gacggacuguauauauauauac......................   3    0    JCK-1
..............................................................................................................................................................................................................gacggacuguauauauauauac......................   3    0    LT-2
..............................................................................................................................................................................................................gacggacuguauauauGuauac......................   1    1    LT-3
..............................................................................................................................................................................................................gacggacuguauauauauauac......................   2    0    JCK-3
..............................................................................................................................................................................................................gacggaUuguauauauauauac......................   1    1    JCK-2
..............................................................................................................................................................................................................gacggacugAauauauauauac......................   3    1    JT-2
..............................................................................................................................................................................................................gacggacuguauauauGuauaca.....................   1    1    LT-3
..............................................................................................................................................................................................................gacggacuguauauauauauaca.....................   1    0    LT-2
..............................................................................................................................................................................................................gacggacuguauauauauauaca.....................   1    0    JCK-1
..............................................................................................................................................................................................................gacggacuguauauauauauaca.....................   2    0    JCK-2
...............................................................................................................................................................................................................acgAacuguauauauauaua.......................   3    1    JT-2
...............................................................................................................................................................................................................acggacuguauaCauauaua.......................   2    1    JCK-3
...............................................................................................................................................................................................................acgAacuguauauauauaua.......................   6    1    JT-3
...............................................................................................................................................................................................................acggacuguauauauauauac......................   1    0    JCK-1
...............................................................................................................................................................................................................acggacuguauauauauaAac......................   1    1    LCK-3
...............................................................................................................................................................................................................acggacuguauauauauauac......................   9    0    JCK-2
...............................................................................................................................................................................................................acggacuguauauauauauac......................   1    0    LT-3
...............................................................................................................................................................................................................acggacuguauauauauauac......................   6    0    JCK-3
................................................................................................................................................................................................................cggacuguauauauauauU.......................   1    1    JCK-1
................................................................................................................................................................................................................cggacuguauauauauauU.......................   2    1    LT-2
................................................................................................................................................................................................................cggaUuguauauauauaua.......................   1    1    LT-2
................................................................................................................................................................................................................cggacuguauauauauauU.......................   1    1    JCK-3
................................................................................................................................................................................................................cgAacuguauauauauaua.......................   1    1    JCK-3
................................................................................................................................................................................................................cggacuguauauauauauU.......................   2    1    JCK-2
................................................................................................................................................................................................................cggacuCuauauauauaua.......................   1    1    LCK-3
................................................................................................................................................................................................................cggacuguaCauauauauac......................   1    1    JCK-2
................................................................................................................................................................................................................cggacuguauauauauauUc......................   1    1    LT-3
................................................................................................................................................................................................................cggacuguauauauauauac......................   1    0    JCK-3
................................................................................................................................................................................................................cggacuguauauauGuauac......................   3    1    LT-2
```
